# Association between statin treatment and new-onset diabetes mellitus: a population based case–control study

**DOI:** 10.1186/s13098-019-0427-9

**Published:** 2019-04-22

**Authors:** Dong-Won Kim, Do-Hoon Kim, Joo-Hyun Park, Moonyoung Choi, Shinhye Kim, Hyonchong Kim, Da-eun Seul, Soo-Gyeong Park, Jin-Hyung Jung, Kyungdo Han, Yong-Gyu Park

**Affiliations:** 1 Department of Family Medicine, Korea University Ansan Hospital, Korea University College of Medicine, 123, Jeokgeum-ro, Danwon-gu, Ansan-si, Gyeonggi-do 15355 Republic of Korea; 2 Department of Biostatistics, Catholic University College of Medicine, Seoul, Republic of Korea

**Keywords:** Statins, New-onset diabetes, Diabetes mellitus, Case–control study, Population-based study

## Abstract

**Background:**

Several studies suggest that statin may increase the risk of new-onset diabetes mellitus (NODM). This study aimed to evaluate the association between the duration and recent use of statin, and the risk of NODM, based on population-based data sets.

**Methods:**

We used the South Korean National Health Insurance Service National Sample Cohort database for this nationwide case–control study. Of the 1 million participants, 6417 participants with NODM in 2012–2013 and 32,085 controls without diabetes (1:5 propensity score matched with age, sex, index year, and year of diabetes diagnosis) were included. In these patients, we examined the statin prescription record for 3 years preceding the outcome. We used conditional logistic regression to calculate the odds ratios (ORs) and 95% confidence intervals (CIs).

**Results:**

After adjustment of covariates, there were no significant differences in the risk of NODM when analyzed according to cumulative use days. The risk of NODM was increased only in the short-term and recent user group (OR 1.48, 95% CI 1.21 to 1.82) whose cumulative prescription days are less than 6 months and whose last prescription is within 6 months of diagnosis.

**Conclusions:**

The risk of NODM was not associated with an increase in the cumulative duration of statin use or with non-recent use. Only recent short-term use of statin was associated with an increased risk of NODM. Diabetes screening are warranted during initial statin therapy.

## Background

Statins, also known as HMG-CoA reductase inhibitors, are among the most commonly used drugs for the prevention of cardiovascular disease (CVD). Clinical evidence for the benefit of statin therapy has already been demonstrated in several studies [1–3], and the drug is generally considered to be relatively safe [4]. Furthermore, in the recent recommendation for the treatment of hyperlipidemia, the 2013 American College of Cardiology/American Heart Association (ACC/AHA) guideline [5] extended the range of statin therapy in comparison to the previous 2002 National Cholesterol Education Program—Adult Treatment Panel III (NCEP-ATP III) guideline [6]. Because of this change, statins are expected to be used more widely.

However, as statin use increases, the risks associated with statin use are important. Questions on the relationship between statin use and new-onset diabetes mellitus (NODM) have been consistently raised, and recently conducted studies have reported several results on the increased risk of NODM [7–12]. Recently, the US Food and Drug Administration issued a warning on the possible increase in glucose and HbA1c levels, through changes in the labeling requirement for statin. In addition, the European Medicines Agency mentioned that statins could increase the risk of type 2 diabetes [13].

However, current available evidence is mainly based on post hoc analyses of randomized controlled trials or meta-analytic results derived from predominantly Western populations. Several studies suggest that Asians are more sensitive to statin therapy and adverse effects could be greater [14, 15], but only a small number of Asians were included in previous studies. Namely, the debate on the side effects of statin-induced diabetes mellitus (DM) is on-going and clear assessment of the increased risk of NODM due to statin use is very important.

Therefore, this study attempted to analyze the risk of NODM according to the duration and recent use of statin therapy using data from the database of the Korean National Health Insurance Service-National Sample Cohort (NHIS-NSC).

## Methods

### Data source

This study used data from the NHIS-NSC database, which consists of approximately one million medical insurance subscribers, who were selected using the stratified random sampling method with 1476 strata by sex (2 strata), age (18 strata), and level of income (41 strata) [16]. NHIS-NSC database is a randomized sample of 2% of the national population of the Republic of Korea and the subscribers were followed from January 2012 to December 2013.

The NHIS-NSC contains information about participants’ insurance eligibility, medical treatment history, healthcare provider’s institution and general health examination. The insurance eligibility database also includes information on the participant’s identity and socioeconomic situation. The medical treatment database includes details of medical treatment, disease diagnoses codes, and prescriptions. The institutional review board (IRB) of Korea University approved the progress of this study (IRB-AS15103). The ethics committee waived the need for participant consent, because the study involved routinely collected medical data that were anonymized at all stages, including during the data cleaning and statistical analysis. This study was carried out according to the ethical principles of the Declaration of Helsinki of the World Medical Association.

### Case–control patient selection from the cohort data

**Fig. 1:**
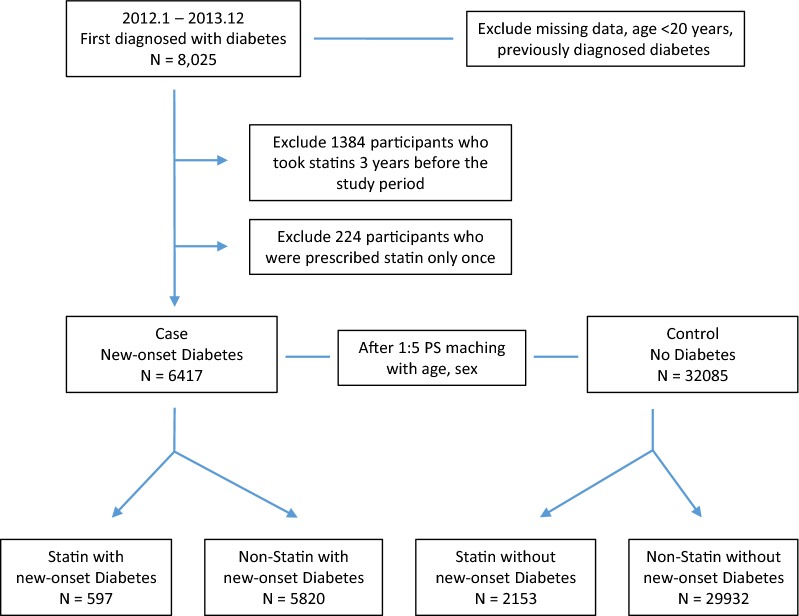
Flow diagram of participants included or excluded

Figure 1 provides information on the participants’ selection. Of the 1 million individuals included in the NHIS-NSC, 387,683, who received screening within the period from January 1, 2012 to December 31, 2013, were selected. A total of 8025 patients who were newly diagnosed with diabetes mellitus (DM), were selected for the study, except for those who were under 20 years of age and those who were prescribed statin only once. In these patients, we observed a record of statin prescriptions for 3 years prior to diagnosis of NODM. Therefore, the study period was defined as 2009–2013, and the wash-out period was 3 years before the study period (2006–2009). We restricted statin users to new statin-users by excluding patients with statin use records for the wash-out period. Patients were also excluded from the study if they were diagnosed with T2DM or they had a history of antidiabetic medication use before the study period. NODM was defined as a fasting glucose level of 126 mg/dl or more, or as a record of a T2DM diagnosis (E11.0–E11.9 based on the ICD-9) and prescription of one or more antidiabetic agents. Finally 6417 patients were selected and assigned to the NODM (case) group. The controls who were not diagnosed for diabetes from 2012 to 2013, were also obtained from the NHIS-NSC database. Of them, 32,085 were assigned to the control group through 1:5 propensity score (PS) matching with age and sex. A PS analysis was carried out on sampled cohorts with logistic regression by age and sex to address selection bias and the presence of potential confounding variables.

### Statin exposure, duration, and recent use

Information on statins was extracted from the NHIS-NSC prescription data. We examined prescription records of statins for the previous 3 years from the date of diagnosis of NODM. Patients with statin prescription record for 3 years before diabetes diagnosis were defined as statin users and patients without prescription records for the same period were defined as statin non-users. The total number of days of administration was obtained in the statin user group. The ICD-9 code confirmed the diabetes diagnosis. In addition, we defined the recent use as the presence of statin prescription within 6 months of outcome, and performed a subgroup analysis, depending on whether the recent use.

### Determinants of disease and demographic factors

The characteristics of the subjects were defined as information from 2012 to 2013 when they were screened. Smoking, drinking, and exercise status were measured by a self-questionnaire and defined as follows; smoking (current smoker, more than 100 cigarettes and current smoker; non-smoker and ex-smoker, less than 100 cigarettes in a lifetime; ex-smoker, more than 100 cigarettes but former smoker), physical activity (regular exercise, 3 or more days of intense 20 min daily workouts, or 5 or more days of moderate 30 min daily workouts, weekly; no regular exercise), Current drinker (current drinker; non-drinker, those who answered “I do not drink” to the question “How many drinks do you drink?”) Socioeconomic status was divided into 2 groups: 70% including upper and middle levels, and 30% including lower levels. Body mass index (BMI) was defined as body weight divided by height squared, and is expressed in units of kg/m^2^ The medical records and diagnoses of the participants were examined for ICD-9 diagnostic codes, drug codes, prescription drugs, and past medical history.

### Potential confounders

We performed variable adjustments to select potential confounders. We adjusted for sex, alcohol consumption, smoking and exercise status, BMI, total cholesterol, waist circumference, and hypertension, which are potential confounders that may affect the relationship between statin use and the generation of diabetes.

### Statistical analysis

Continuous variables are compared using paired t-tests, and categorical variables are compared using Chi-square tests. A conditional logistic regression model was used to estimate the relative importance of statin therapy. Five groups were identified according to the duration of statin administration: (1) non-users, (2) less than 6 months, (3) 6 months to less than 1 year, (4) 1 year to less than 2 years, (5) 2 year to less than 3 years. For the subgroup analysis of recent use, we divided participants into two groups: recent users (with a prescription record within 6 months) and others (no prescription record within 6 months). Odds ratios (ORs) and 95% confidence intervals (CIs) were calculated using unexposed patients as a reference. All statistical analyses were performed using the SAS statistical package (version 9.3, SAS Institute INC). P < 0.05 was considered statistically significant in all the tests.

## Results

Table 1 shows the baseline characteristics of those with NODM (case group) and with the non-diabetes control group. We compared each characteristic between the groups, and the 2 groups were balanced in age and sex. The NODM group had the higher BMI, waist circumference (WC), systolic BP (SBP), diastolic BP (DBP), low-density lipoprotein-cholesterol (LDL-C) and triglyceride (TG) level and lower high-density lipoprotein-cholesterol (HDL-C) level than those of non-NODM group. NODM group is more likely to smoke cigarettes and exercise, and less drink alcohol. Socio-Economic levels were lower in the NODM group.

**Table 1:** Participants Characteristics

	Total	Cases	Controls	P value
	N = 38,502	n = 6417	n = 32,085	
Age, year	51.1 ± 13.1	51.1 ± 13.2	51.1 ± 13.1	0.99
Age ≥ 60 year	9493 (24.7)	1581 (24.6)	7912 (24.7)	0.97
Male	26,050 (67.7)	4342 (67.7)	21,708 (67.7)	0.99
Statin use	2750 (7.1)	597 (9.3)	2153 (6.7)	< 0.0001
Recent statin use within 6 months	1887 (4.9)	439 (6.8)	1448 (4.5)	< 0.0001
BMI, kg/m^2^	23.9 ± 3.2	25.2 ± 3.8	23.7 ± 3.1	< 0.0001
BMI > 25 kg/m^2^	13,222 (34.3)	3117 (48.6)	10,105 (31.5)	< 0.0001
Waist circumference, cm	81.7 ± 8.9	84.8 ± 9.4	81.1 ± 8.6	< 0.0001
SBP, mmHg	123.5 ± 14.9	128.2 ± 15.6	122.6 ± 14.6	< 0.0001
DBP, mmHg	77.2 ± 10.1	80 ± 10.3	76.7 ± 10	< 0.0001
HDL-cholesterol, mg/dL	54.1 ± 16.6	53 ± 18	54.4 ± 16.3	< 0.0001
LDL-cholesterol, mg/dL	115.7 ± 32.6	117 ± 37.3	115.4 ± 31.5	0.0004
Triglyceride, mg/dL	139.9 ± 97	180.4 ± 126.2	131.9 ± 87.9	< 0.0001
Current smoker	11,477 (29.8)	2273 (35.4)	9204 (28.7)	< 0.0001
Current drinker	21,277 (55.3)	17,532 (54.6)	3745 (58.4)	< 0.0001
Regular exercise^a^	6690 (17.4)	5702 (17.8)	988 (15.4)	< 0.0001
Low social economic status	5081 (13.2)	1000 (15.6)	4081 (12.7)	< 0.0001
Hypertension				

Data reported as n (%) or mean ± SD

*BMI* body mass index, *SBP* systolic blood pressure, *DBP* diastolic blood pressure, *HDL* high-density lipoprotein, *LDL* low-density lipoprotein

^a^Intensive exercise 3 days or more per week, or moderate exercise 5 days or more per week

Table 2 shows the association of NODM risk with the duration of statin therapy. After PS matching and adjusting for age and sex, the risk of NODM was significant in statin users compared with non-statin users. However after additional adjustment for drinking, smoking, exercise, BMI, HDL-C, LDL-C, TG, WC, hypertension, no significant increase in NODM risk was observed in the statin user during the 3-year observation period. When analyzed on a per-term basis, significant results were obtained between statin treatment and risk of NODM after the adjustment for age and sex, but were not showed after the adjustment for multiple covariates regardless of the duration of the therapy.

**Table 2:** Associations of new-onset diabetes mellitus risk according to cumulative duration of statin therapy

	No statin use	Statin use	Cumulative duration of statin therapy			
			< 6 months	6 months–1 year	1–2 year	2–3 year
Number (%)	35,752 (100)	2750 (100)	1175 (41.9)	626 (23.16)	645 (24.08)	304 (10.87)
Model 1* Odds ratio (95% CI)	1 (Reference)	1.44 (1.31,1.59)	1.54 (1.34,1.77)	1.46 (1.20,1.77)	1.28 (1.05,1.55)	1.39 (1.05,1.83)
Model 2** Odds ratio (95% CI)	1 (Reference)	1.03 (0.93,1.14)	1.11 (0.95,1.29)	1.02 (0.84,1.25)	0.90 (0.74,1.11)	1.06 (0.79,1.41)

*Model 1 was adjusted for age and sex

**Model 2 was adjusted for age, sex, drinking, smoking, regular exercise, body mass index, high-density lipoprotein-cholesterol, low-density lipoprotein-cholesterol, triglyceride, waist circumference and hypertension

Namely, no statistically significant difference was found in the simple comparison of statin and NODM (OR 1.03, 95% CI 0.93 to 1.14). Analysis of the treatment duration also did not show an increase in NODM risk in all the groups. This implies that the duration of statin therapy is not associated with an increased risk of NODM.

This study further analyzed the effect of recent statin use on NODM risk. Table 3 shows an analysis of the increased risk of NODM with statin therapy duration and recent use. After adjustment for covariates, NODM risk was statistically significantly increased in patients who were prescribed statins for a short period of less than 6 months and within the last 6 months compared to non-statin users (OR 1.48, 95% CI 1.21 to 1.82).

**Table 3:** Subgroup analysis of statin therapy duration and recent statin use

	Model 1* Odds ratio (95% CI)	Model 2** Odds ratio (95% CI)
No statin use	1 (Reference)	1 (Reference)
Cumulative duration < 6 months		
Recent use within 6 months***	2.04 (1.69,2.47)	1.48 (1.21,1.82)
No recent use	1.17 (0.95,1.43)	0.82 (0.65,1.02)
Cumulative duration ≥ than 6 months		
Recent use within 6 months***	1.41 (1.23,1.61)	1.0 (0.87,1.15)
No recent use	1.15 (0.82,1.62)	0.86 (0.61,1.23)

*Model 1 was adjusted for age and sex

**Model 2 was adjusted for age, sex, drinking, smoking, regular exercise, body mass index, high-density lipoprotein-cholesterol, low-density lipoprotein-cholesterol, triglyceride, waist circumference and hypertension

***Recent use was defined as the presence of statin prescription within 6 months of outcome

## Discussion

In the present study, the risk of NODM did not increase as the cumulative number of days of statin use increased. Also, the risk of NODM did not increase in non-recent statin users. We observed that the risk of NODM increased only in those in the recent users group who received statin within the last 6 months and short-term user group in the duration of less than 6 months. Namely, the risk of NODM increased in the early stages of statin treatment in Korean general population. This study was conducted to obtain clinically valuable results by analyzing how NODM risk differs according to the time and interval of taking medicine.

The results of the previous studies on the associations between statin use and NODM were as follows. In the Justification for the Use of Statins in Primary Prevention: An Intervention Trial Evaluating Rosuvastatin (JUPITER) trial [17], which demonstrated the efficacy of rosuvastatin in preventing CVD, a 25% increase in diabetes risk was reported. In the Anglo-Scandinavian Cardiac Outcomes Trial–Lipid Lowering Arm (ASCOT–LLA) [18], atorvastatin reduced incidences of non-fatal myocardial infarction and fatal coronary artery disease by 36%, and increased the risk of diabetes by 14%. Most other studies and several meta-analyses have also reported that statin use increases the risk of NODM [8, 11, 19–23]. These studies have reported that statin increases NODM regardless of the type, although there is a difference in degree.

One WOSCOPS study reported that men with hypercholesterolemia, aged 45–64 years, had a 30% reduction in the risk of developing NODM after 5 years of pravastatin treatment [24]. However, in the PROSTPER study where pravastatin was given to elderly subjects aged 70 years or older, the risk of diabetes increased by 32% by statin treatment [22].

Since statin has been implicated in the development of NODM, several studies have reported the biologic mechanisms behind the diabetes-inducing effects of statin. One study suggests that the inhibition of the statin target 3-hydroxy-methylglutaryl-CoA reductase (HMGCR) is an important mechanism [25]. HMGCR genetic variants and statin treatment were associated with higher body weight and higher risk of type 2 diabetes, suggesting that these effects are a consequence of HMGCR inhibition [25]. In addition, several independent studies reported that low LDL-C levels have been associated with an increased risk of NODM [26]. The genetic predisposition of dyslipidemia, FBG, HbA1c, and HOMA-IR, was associated with a lower level of diabetes-related indicators [27]. High blood LDL-C levels were also associated with a lower risk of diabetes, which was demonstrated by an SNP analysis of the lipid metabolism-related genes [28]. Another study also reported that the prevalence of type 2 DM was low in patients with familial hypercholesterolemia [29]. As another mechanism, several studies have shown that statin therapy can be detrimental to pancreatic beta cell function [30]. Statin dose-dependently induces ß cell damage and insulin resistance in the smooth muscle cells [31], reduces glucose transporter 4 (GLUT4) expression which is involved in glucose uptake in the peripheral cells [31–33], and decreases insulin signaling [34, 35]. It also inhibits adipocyte differentiation and leads to cell accumulation, so the insulin-sensitive and insulin-resistance hormones cannot be secreted [32]. Further studies are underway, focusing on the additional mechanisms such as the link between statin therapy and specific microRNAs associated with reduced insulin secretion [30].

As such, many studies have shown that the use of statin increases the NODM risk [8–12, 36–38]. However, very few studies have been made on the effects of recent statin uses on the incidence of diabetes, particularly in general Asian populations. Several reports have suggested that recent statin dosing may increase the risk of NODM. There are reports that the short-term risk of diabetes due to statin therapy is higher in patients with diabetes risk factors [37, 39, 40]. In prediabetes patients, who are susceptible to the development of type 2 diabetes, the effects of statin use, such as insulin resistance induction, decreased GLUT4 expression and pancreatic β cell function, may contribute to an increased risk of diabetes [30–35]. In addition, recent guidance suggests that checking FBG and HbA1c levels before starting statin treatment may be helpful in pre-diabetic patients with high diabetes risk [41]. This suggests that the results of the present study support the clinical significance of diabetes screening in patients treated with statins.

This study has several strengths. In the present study, we used NHIS-NSC data to include various factors such as age, sex, alcohol consumption, smoking and exercise status, BMI, HDL-C, LDL-C and TG level, WC and hypertension. Calibration improved the quality of the analysis. We also analyzed the association between statin and NODM risk based on a large-scale database of 1 million people. This study is based on the cohort data of 2% of the population of the Republic of Korea that is representative of the nationwide population. Unlike previously conducted retrospective studies, in this study, each patient was analyzed for NODM risk according to statin use for the same 3-year period. PS matching is a statistically meaningful analytical technique that can effectively control disturbance factors. In this research, we strived to minimize statistical bias through PS matching. We also presented a new perspective on the relationship between statin and NODM risk, by analyzing the duration and recent use of statin therapy, which were previously untried.

Nonetheless there are some limitations to this study. First, the NHIS-NSC database does not contain HbA1c results, so it is hard to tell if the definitive diagnostic criteria for diabetes are applied. Second, we could not consider the type of statin and dosage. A meta-analysis has shown that intensive-dose statin therapy was associated with an increased risk of NODM compared with moderate-dose statin therapy [42]. Further research is therefore needed.

There is a possibility of an indication bias between the recent use of statin and increased risk of NODM, as with other observational studies. Prediabetes, an important risk factor for type 2 diabetes, is often associated with hyperlipidemia [43]; therefore, it is highly likely that participants with prediabetes had been treated with statin. There is also the possibility of detection bias. It is possible to speculate that patients who have been prescribed statins are clinically evaluated because of the high frequency of visits to the hospital, and are likelier to be diagnosed with diabetes. Unlike randomized studies, observational studies are based on long-term follow-up data from large numbers of participants, which may increase the chance of developing or diagnosing diseases such as diabetes, which can take many years. In this study, we tried to adjust the parameters to eliminate the above bias through PS matching.

## Conclusion

This nationwide survey using medical claim data from the NHIS-NSC has shown that the risk of NODM did not increase as the cumulative number of days of statin use increased. Also, the risk of NODM did not increase in non-recent statin users. The risk of NODM increased only in the early stages of statin treatment in Korean general population.

This study is valuable in terms of supporting the clinical value of performing diabetes screening, clinically, when starting statin therapy. When starting statin therapy, life style modification needs to be emphasized and the potential benefits and side effects of statin need to be discussed. Also, periodic screening and monitor-ing for DM may be required. In addition, these findings warrant further studies to determine how long and at what intervals diabetes screening should be initiated after starting statin therapy.
